# Adenoviral vectors encoding CRISPR/Cas9 multiplexes rescue dystrophin synthesis in unselected populations of DMD muscle cells

**DOI:** 10.1038/srep37051

**Published:** 2016-11-15

**Authors:** Ignazio Maggio, Jin Liu, Josephine M. Janssen, Xiaoyu Chen, Manuel A. F. V. Gonçalves

**Affiliations:** 1 Leiden University Medical Center, Department of Molecular Cell Biology, Einthovenweg 20, 2333 ZC, Leiden, the Netherlands

## Abstract

Mutations disrupting the reading frame of the ~2.4 Mb dystrophin-encoding *DMD* gene cause a fatal X-linked muscle-wasting disorder called Duchenne muscular dystrophy (DMD). Genome editing based on paired RNA-guided nucleases (RGNs) from CRISPR/Cas9 systems has been proposed for permanently repairing faulty *DMD* loci. However, such multiplexing strategies require the development and testing of delivery systems capable of introducing the various gene editing tools into target cells. Here, we investigated the suitability of adenoviral vectors (AdVs) for multiplexed *DMD* editing by packaging in single vector particles expression units encoding the *Streptococcus pyogenes* Cas9 nuclease and sequence-specific gRNA pairs. These RGN components were customized to trigger short- and long-range intragenic *DMD* excisions encompassing reading frame-disrupting exons in patient-derived muscle progenitor cells. By allowing synchronous and stoichiometric expression of the various RGN components, we demonstrate that dual RGN-encoding AdVs can correct over 10% of target *DMD* alleles, readily leading to the detection of Becker-like dystrophin proteins in unselected muscle cell populations. Moreover, we report that AdV-based gene editing can be tailored for removing mutations located within the over 500-kb major *DMD* mutational hotspot. Hence, this single *DMD* editing strategy can in principle tackle a broad *spectrum* of mutations present in more than 60% of patients with DMD.

Duchenne muscular dystrophy (DMD), affecting ∼1 in 4,000 newborn boys^1^, is amongst the most severe and common forms of muscular dystrophies, a heterogeneous group of inherited disorders marked by progressive muscle weakness and wasting^2^,^3^. The molecular basis of DMD, known since 1987, features different loss-of-function mutations within the ~2.4 Mb dystrophin-encoding *DMD* gene (Xp 21.2) (ref. 4). Although duplications and point mutations give rise to this pathology, the vast majority of DMD-causing mutations consists of intragenic deletions, comprising one or more exons^5^. Of note, genomic defects located in a major mutation prone “hotspot” region, spanning exons 45 through 55, account for more than 60% of the population of patients with DMD^5^. Regardless of their nature and location, most DMD-causing mutations lead to reading frame disruptions resulting in a lack of dystrophin, an essential musculoskeletal protein. Amongst other functions, this rod-shaped protein is involved in muscle cell stability since it provides a key link between the dystrophin-associated protein complex (DAPC) embedded in the sarcolemma and the actin mesh in the cytoskeleton. Hence, in patients with DMD, dystrophin deficiency compromises the integrity of muscle cells ultimately resulting in progressive skeletal and cardiac myopathy leading to fatality, normally between the second and third decades of life^2^,^6^. Despite many research efforts, to date DMD treatments remain palliative and supportive rather than curative^2^,^6^,^7^.

Gene therapy is being pursued as a potential DMD therapeutic option whose benefits may be maximized if combined with pharmacological and cell-based approaches. Notably, the very large size (i.e. ~11 kb) of the full-length *DMD* coding sequence (CDS) (refs 2 and 6) puts it outside the packaging capacity of most commonly used viral vectors such as that of ∼4.7 kb of recombinant adeno-associated virus (rAAV) particles^8^. It is also known that in-frame deletions within *DMD*, resulting in internally truncated dystrophins, give rise to Becker muscular dystrophy (BMD), a less severe form of muscular dystrophy. This observation has led to the investigation of therapeutic interventions based on gene replacement and exon skipping^2^,^6^,^7^. The former entails the delivery of dystrophin lacking non-essential domains (e.g. microdystrophins), while the latter exploits the use of antisense oligonucleotides for interfering with the splicing machinery and prevent the inclusion of reading frame-disrupting exons into the *DMD* mRNA. Additionally, programmable nuclease-assisted genome editing has been put forward as yet another potential therapeutic modality that, by directly correcting defective *DMD* loci, assures permanent dystrophin synthesis from their native regulatory elements^9^,^10^,^11^,^12^,^13^.

RNA-guided nucleases (RGNs) constitute particularly powerful genome editing tools^14^,^15^,^16^. The most commonly employed RGNs are based on the type II clustered, regularly interspaced, short palindromic repeats (CRISPR)-associated Cas9 (CRISPR-Cas9) system from *Streptococcus pyogenes*. In this binary ribonucleoprotein complex, a single guide RNA (gRNA) addresses the Cas9 nuclease to predefined genomic sequences^17^. These target sequences consist of 18–20 nucleotides, complementary to the 5′ end of the gRNA, followed by a protospacer adjacent motif (PAM; NGG, in the case of *S. pyogenes* Cas9) (ref. 17). The Cas9 generates a blunt-ended double-stranded DNA break (DSB) that triggers endogenous DNA repair pathways which are ultimately exploited for achieving permanent and targeted genetic modifications^13^. In mammalian cells, a major DNA repair pathway is that of non-homologous end joining (NHEJ). This pathway culminates with a direct end-to-end ligation of DNA termini often resulting in the incorporation of small insertions and deletions (indels) (ref. 13).

An appealing feature of RGNs is their versatility for multiplexing purposes. Related to this, it has been shown that expressing two or more gRNAs addressing Cas9 to different genomic sites, enables targeting multiple genes or triggering genomic alterations between pairs of DSBs such as intragenic deletions^13^,^14^,^18^. Of note, proof-of-principle studies have demonstrated that such RGN multiplexes can be employed to remove reading frame-disrupting exons from *DMD* loci^19^. These manoeuvres result in the expression of in-frame mRNA transcripts which are translated into shorter, but still functional, dystrophins which are reminiscent of those underlying mild BMD phenotypes. Such multiplexing strategies have been validated in patient-derived myoblasts^19^,^20^, induced pluripotent stem cells (iPSCs) (ref. 21) and dystrophic Dmd^*mdx*^ mice^22^,^23^,^24^,^25^. Of note, experiments performed in iPSCs and human myoblasts have demonstrated that RGN multiplexes can also trigger the deletion of the major *DMD* mutational hotspot^19^,^20^,^21^. These studies provide a strong rationale for developing DMD therapeutic strategies based on RGN multiplexes. However, more research is needed especially for achieving simultaneous, uniform and efficient introduction of all nuclease components into target cells.

In the present work, we sought to investigate the feasibility and suitability of using “all-in-one” adenoviral vectors (AdVs) for delivering RGN multiplexes designed for removing single or multiple exons from defective *DMD* alleles. Following AdV transductions of patient-derived muscle progenitor cells, we report that these NHEJ-mediated gene editing strategies result in efficient *DMD* reading frame restoration and yield readily detectable dystrophin synthesis in muscle cells differentiated from unselected target cell populations. Notably, we demonstrated that “all-in-one” AdVs can mediate the removal of mutations located within the over 500-kb major *DMD* mutational hotspot allowing for tackling more than 60% of the registered *DMD* mutations^5^ via a single viral-based gene editing strategy. Furthermore, we tested side-by-side *DMD* editing based on “all-in-one” AdV delivery of dual and single RGN complexes. The latter strategies are customized to correct specific *DMD* mutations via NHEJ-mediated reading frame resetting and DNA-borne exon skipping^20^. We demonstrate that AdVs encoding single or dual RGN complexes can lead to *DMD* repair and *de novo* dystrophin protein synthesis without having to resort to cell selection schemes. Collectively, this study establishes AdVs as a versatile and powerful platform for the delivery of RGN multiplexes into therapeutically relevant cells.

## Results

### Packaging RGN multiplexes in single AdV particles

**Figure 1:**
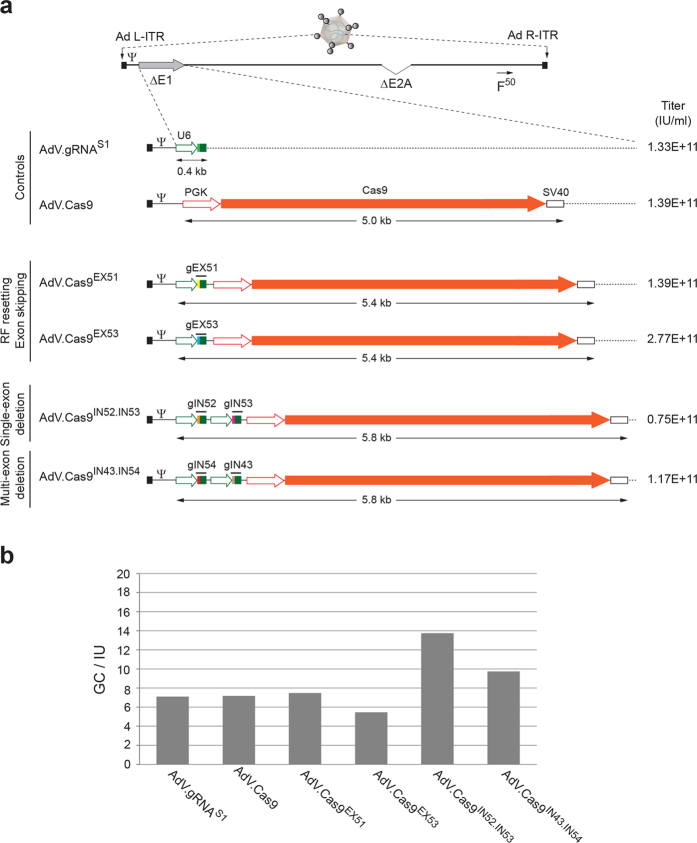
Design and generation of AdVs encoding RNA-guided nuclease components. (**a**) Schematic representation of the genome structure of control and *DMD*-targeting AdVs. The genomes of dual RGN-encoding AdV.Cas9^IN52.IN53^ and AdV.Cas9^IN43.IN54^ are depicted in relation to those of single RGN-encoding AdV.Cas9^EX51^ and AdV.Cas9^EX53^. The AdV.gRNA^S1^ and AdV.Cas9, expressing exclusively an irrelevant gRNA and Cas9, respectively, served as controls. The AdV.Cas9^EX51^ and AdV.Cas9^EX53^ particles code for Cas9:gEX51 and Cas9:gEX53 complexes, respectively. The target sites of Cas9:gEX51 and Cas9:gEX53 complexes are located in exon 51 and exon 53 of the *DMD* locus, respectively. AdV.Cas9^IN52.IN53^ and AdV.Cas9^IN43.IN54^ deliver a pair of *gRNA* cassettes together with a *Cas9* transcriptional unit. The gRNAs gIN52 and gIN53 address the Cas9 nuclease to *DMD* sequences in intron 52 and intron 53, respectively; the gRNAs gIN43 and gIN54 target the same nuclease to sequences located in intron 43 and intron 54, respectively. All vectors deployed in this study were isogenic in the sense that they were all assembled on the basis of a second-generation (i.e. *E1*- and *E2A*- deleted) AdV backbone coding for chimeric fibers (F^50^) consisting of basal domains from human adenovirus serotype-5 and apical shaft and knob motifs from human adenovirus serotype-50. Each *gRNA* expression unit is under the transcriptional control of RNA Pol-III promoter (i.e. U6) and terminator sequences, whereas the *Cas9* ORF is under the transcriptional control of the human *PGK-1* promoter (PGK) and the simian virus 40 polyadenylation signal (SV40). Ψ, human adenovirus serotype-5 packaging signal; Ad L-ITR and Ad R-ITR, “left” and “right” adenoviral inverted terminal repeat, respectively; RF, reading frame (**b**) Ratios of genome copies (GC) to infectious units (IU). The GC and IU concentrations in purified preparations of the indicated AdVs were determined by using fluorometric and TCID_50_ assays, respectively.

We started by investigating the suitability of AdV particles for packaging DNA coding for Cas9 and gRNA pairs targeting intronic *DMD* sequences. To this end, we selected RGN complexes customized to trigger short- and long-range intragenic *DMD* excisions after the activation of the NHEJ pathway^20^. In particular, the gRNA pair gIN52 and gIN53 was designed to induce a short, mutation-specific, *DMD* excision spanning exon 53. Next to this, the gRNA pair gIN43 and gIN54 was customized to tackle a large range of *DMD* mutations by triggering DSB formation in introns 43 and 54 flanking the major *DMD* mutational hotspot. Thus, in the current work, we generated AdV.Cas9^IN52.IN53^ and AdV.Cas9^IN43.IN54^ particles containing expression units encoding the aforementioned gRNA pairs and the *S. pyogenes* Cas9 protein (Fig. 1a). Importantly, these second-generation (i.e. *E1*- and *E2A*- deleted) AdVs display apical fiber motifs from adenovirus serotype-50 (F^50^) (Fig. 1a). In contrast to fibers from prototypic adenovirus serotype-5, that bind the coxsackievirus-adenovirus receptor (CAR), the F^50^ motifs recognize the cellular receptor CD46 instead. This allows fiber-modified AdVs to bypass the absence of CAR on the surface of therapeutically relevant cells, including human muscle progenitor cells and non-muscle cells with myogenic capacity^20^,^26^,^27^,^28^.

After rescuing, propagating and purifying the dual RGN-encoding AdV particles (i.e. AdV.Cas9^IN52.IN53^ and AdV.Cas9^IN43.IN54^), we determined that their functional titers and genome copies-to-infectious units ratios were in the range of those of isogenic AdV controls. These controls encode exclusively Cas9, an irrelevant gRNA or encode Cas9 together with a single *DMD*-targeting gRNA (Fig. 1b). In addition, restriction fragment length analysis was performed on DNA isolated from purified AdV.Cas9^IN52.IN53^ and AdV.Cas9^IN43.IN54^ particles. This assay revealed neither large sequence rearrangements nor deletions indicating that both vector genomes were packaged intact (Supplementary Fig. S1). These data demonstrate that tandem gRNA and Cas9 expression units can be stably maintained during the rescue and propagation of AdV particles leading to the packaging of full-length recombinant vector genomes.

### “All-in-one” AdV particles achieve *DMD* repair through short-range, single-exon, excision

**Figure 2:**
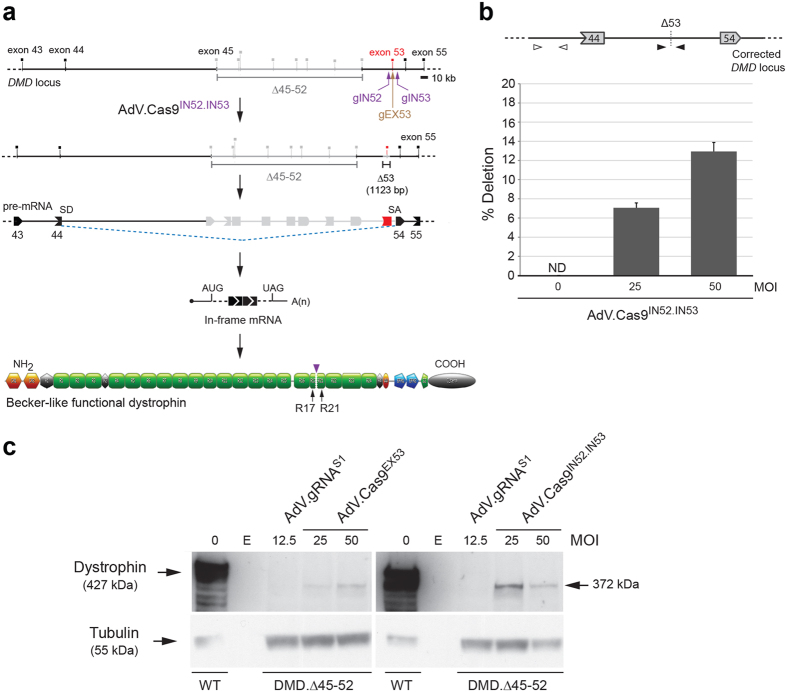
“All-in-one” AdV transduction of paired RGNs for *DMD* repair via short-range intragenic deletions. (**a**) *DMD* repair through NHEJ-mediated exon 53 excisions in DMD.Δ45-52 muscle cells. Genomic deletions spanning exon 45 through exon 52 disrupt the *DMD* reading frame and yield out-of-frame mRNA transcripts carrying a premature stop codon within exon 53 (red box). RGN multiplexes consisting of Cas9 bound to intron-specific gIN52 and gIN53 gRNAs are designed to trigger the excision of a 1,123-bp DNA segment encompassing exon 53. This strategy is expected to induce the synthesis of a Becker-like dystrophin as splicing of exon 44 to exon 54 generate in-frame mRNA transcripts. gEX53, gRNA targeting Cas9 to a sequence within exon 53; SD, splice donor; SA, splice acceptor. Orange hexagons, CH1 and CH2 actin-binding domain 1; black hexagons, hinges; green boxes, spectrin-like repeats; orange oval, WW domain; blue pentagons, EFH1 and EFH2 hand-regions containing cysteine-rich motifs which, amongst other proteins, bind to β-dystroglycan; green pentagon, ZZ zinc finger domain; grey oval, C-terminal domain. Vertical arrowhead, protein junction formed by the *DMD* editing procedure. (**b**) qPCR analysis for determining the frequencies of *DMD* exon 53 deletion. The qPCR assay was applied on genomic DNA from DMD.Δ45-52 myoblasts transduced with “all-in-one” vector AdV.Cas9^IN52.IN53^. The DMD.Δ45-52 myoblasts were transduced with AdV.Cas9^IN52.IN53^ at the indicated multiplicities of infection (MOI). qPCR assays were carried out with primer pairs flanking the RGN-induced intronic junction (solid arrowheads). A primer pair targeting intron 43 sequences (open arrowheads) served as an internal control for determining the amounts of input DNA. MOI, multiplicity of infection; Error bars, standard deviations of technical replicates; ND, not detected. **(c)** Dystrophin western blot analysis after AdV.Cas9^EX53^ and AdV.Cas9^IN52.IN53^ transductions. The DMD.Δ45-52 myoblasts were transduced with AdV.Cas9^EX53^ and AdV.Cas9^IN52.IN53^ at multiplicities of infection (MOI) of 25 and 50 IU/cell. Cultures with myotubes differentiated from mock-transduced wild-type myoblasts (WT) served as positive controls. Negative controls were obtained from cultures containing myotubes differentiated from DMD.Δ45-52 myoblasts transduced at an MOI of 12.5 IU/cell with AdV.gRNA^S1^. After 4 days in differentiation medium, western blot analysis was performed on unselected AdV-transduced muscle cell populations. Tubulin provided for a loading control antigen. E, empty lane.

To investigate AdV-based *DMD* editing by targeted single-exon excision, we used the AdV.Cas9^IN52.IN53^ to transduce patient-derived myoblasts lacking exons 45 through 52 (DMD.Δ45-52). This defective genotype can be tackled by the permanent chromosomal excision of exon 53 since the resulting DNA template generates in-frame mRNA transcripts (Fig. 2a). Of note, approximately 8% to 10% of disrupted *DMD* reading frames identified in the population with DMD can be fixed by this approach^5^,^29^.

To start *DMD* editing experiments, DMD.Δ45-52 myoblasts were either mock-transduced or were transduced with AdV.Cas9^IN52.IN53^ at MOIs of 25 and 50 IU/cell. At 7 days post-transduction, a qPCR assay designed for quantifying the DNA junctions generated after the simultaneous expression of Cas9:gIN52 and Cas9:gIN53 complexes, revealed that AdV.Cas9^IN52.IN53^ mediated permanent *DMD* repair in up to 13% of DMD.Δ45-52 cells (Fig. 2b).

Considering that AdV.Cas9^IN52.IN53^ transductions did not abolish the differentiation and fusion capacity of gene-edited DMD.Δ45-52 myoblasts, we proceeded to assess the rescue of dystrophin synthesis in DMD muscle cell populations. To this end, parallel transduction experiments were performed in DMD.Δ45-52 myoblasts by deploying AdV.Cas9^IN52.IN53^, isogenic AdV.Cas9^EX53^ and, as negative control vector, AdV.gRNA^S1^. Of note, AdV.Cas9^EX53^ delivers a single Cas9:gEX53 complex customized to induce DSBs at a sequence in exon 53, which trigger NHEJ in generating indels that disrupt splicing motifs or shift the reading frame to the proper coding sequence. The resulting DNA-borne exon skipping and reading frame resetting events repair the defective *DMD* locus in DMD.Δ45-52 cells^20^. After triggering myoblast differentiation, western blot analysis revealed the presence of Becker-like dystrophin proteins in cultures of myotubes whose progenitors had undergone AdV.Cas9^EX53^ and AdV.Cas9^IN52.IN53^ transductions (Fig. 2c). As expected, DMD myoblasts exposed to the control vector AdV.gRNA^S1^ did not yield detectable levels of dystrophin once differentiated into myotubes (Fig. 2c). Importantly, based on the relative intensities of the western blot signals diagnostic for dystrophin, the levels of *de novo* dystrophin protein synthesis resulting from AdV.Cas9^IN52.IN53^ transductions were not inferior to those achieved by the isogenic AdV.Cas9^EX53^ (Fig. 2c).

### “All-in-one” AdV particles achieve *DMD* repair through long-range, multi-exon, excision

**Figure 3:**
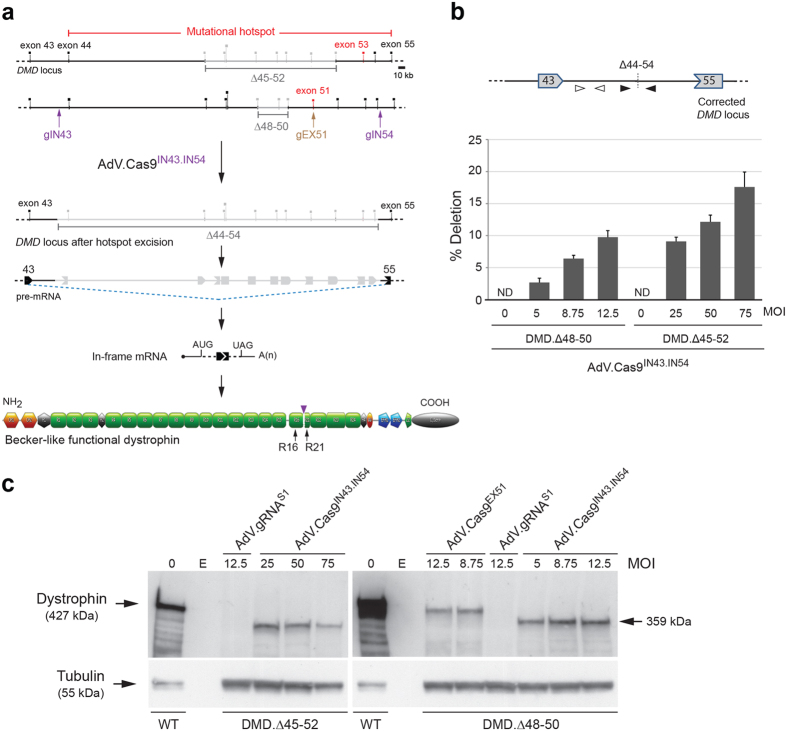
“All-in-one” AdV transduction of paired RGNs for *DMD* repair via long-range intragenic deletions. (**a**) *DMD* repair through NHEJ-mediated long-range, multi-exon, deletion. DMD-causing mutations located in the major *DMD* mutational hotspot (spanning exon 45 through exon 55) can, in principle, be tackled by a single multiplexing strategy based on the excision of a DNA segment encompassing exon 44 through exon 54. This multi-exon deletion strategy can build on the coordinated action of a pair of RGNs, consisting of Cas9 bound to the intron-specific gIN43 or gIN54 gRNAs. These RGNs are designed to repair the *DMD* reading frame as the joining of exon 43 to exon 55 is expected to yield in-frame mRNA species coding for an internally truncated Becker-like dystrophin. gEX51, gRNA targeting Cas9 to a sequence within exon 51. Vertical arrowhead, protein junction formed by the *DMD* editing procedure. For the naming of the dystrophin structural domains see legend of Fig. 2a. (**b**) qPCR analysis for establishing the frequencies of *DMD* multi-exon deletions. The qPCR assay was applied on genomic DNA from DMD.Δ48-50 and DMD.Δ45-52 myoblasts transduced with “all-in-one” vector AdV.Cas9^IN43.IN54^. The DMD.Δ48-50 and DMD.Δ45-52 myoblasts were transduced with AdV.Cas9^IN43.IN54^ at the indicated multiplicities of infection (MOI). qPCR assays were carried out with primer pairs flanking the expected RGN-induced intronic junction (solid arrowheads). qPCR amplifications with primer pair targeting intron 43 sequences (open arrowheads) provided for an internal control to determining the amounts of input DNA. Error bars, standard deviations of technical replicates; ND, not detected. **(c)** Dystrophin western blot analysis after AdV.Cas9^EX51^ and AdV.Cas9^IN43.IN54^ transductions. The DMD.Δ45-52 and DMD.Δ48-50 myoblasts were transduced with AdV.Cas9^IN43.IN54^; the DMD.Δ48-50 myoblasts were transduced with AdV.Cas9^EX51^. The multiplicities of infection (MOI) used are indicated. Cultures with myotubes differentiated from mock-transduced wild-type myoblasts (WT) served as positive controls. Negative controls were obtained from cultures containing DMD.Δ45-52 and DMD.Δ48-50 myotubes whose progenitors had been transduced with AdV.gRNA^S1^ at an MOI of 12.5 IU/cell. After exposing DMD.Δ45-52 and DMD.Δ48-50 myoblasts to differentiation medium for 4 and 5 days, respectively, western blot analysis was performed on unselected AdV-transduced muscle cell populations. Tubulin provided for a loading control antigen. E, empty lane.

To broaden the potential applicability of AdV-based *DMD* editing, we have investigated the suitability of AdV.Cas9^IN43.IN54^ for chromosomal removal of sequences framing the major *DMD* mutational hotspot. To this end, we deployed patient-derived myoblasts with two different *DMD* genotypes (i.e. DMD.Δ48-50 and DMD.Δ45-52) (Fig. 3a). These and all the other faulty genotypes nested within the major *DMD* mutational hotspot region can, in principle, be tackled by AdVs encoding Cas9:gIN43 and Cas9:gIN54 complexes since these RGNs generate DSBs at their cognate sequences in intron 43 and intron 54, respectively. The NHEJ-mediated end-to-end ligation of the resulting chromosomal termini, and concomitant excision of the intervening DNA, is expected to rescue dystrophin synthesis as splicing of exon 43 to exon 55 generates in-frame RNA transcripts (Fig. 3a).

Therefore, we performed *DMD* editing experiments in which DMD.Δ48-50 and DMD.Δ45-52 myoblasts were either mock-transduced or were transduced with AdV.Cas9^IN43.IN54^ at MOIs ranging from 5 to 75 IU/cell. At 7 days post-transduction, a PCR assay with primers flanking the expected genomic excision, readily led to the detection of amplicons diagnostic for the *de novo* generated junction Δ44-54 exclusively in AdV.Cas9^IN43.IN54^-transduced myoblast populations (Supplementary Fig. S2). Importantly, a qPCR assay analogous to that used for measuring single-exon deletions (Fig. 2b), revealed a clear AdV.Cas9^IN43.IN54^ dose-dependent increase in *DMD* editing frequencies in DMD.Δ48-50 and DMD.Δ45-52 target cell populations (Fig. 3b). The fractions of *DMD*-edited myoblasts ranged from a minimum of 3% to a maximum of 18% (Fig. 3b). Interestingly, these gene correction levels were similar to those achieved in parallel transduction experiments in which isogenic AdV.Cas9^EX51^ particles were used instead for targeted *DMD* repair in DMD.Δ48-50 myoblasts (Supplementary Fig. S3). In contrast to AdV.Cas9^IN43.IN54^, isogenic AdV.Cas9^EX51^ works analogously to AdV.Cas9^EX53^ in that it delivers a single RGN complex (i.e. Cas9:gEX51) customized to repair the defective *DMD* locus via DNA-borne exon skipping and reading frame resetting^20^. Therefore, this side-by-side comparison of *DMD* editing following AdV.Cas9^IN43.IN54^ and AdV.Cas9^EX51^ transductions, suggests that delivering RGN multiplexes, instead of a single RGN complex, in AdV particles is not, *per se,* a limiting factor for achieving efficient endogenous *DMD* repair.

Subsequently, we investigated the capacity of AdV.Cas9^IN43.IN54^ to induce robust *de novo* dystrophin synthesis in bulk populations of differentiated myoblasts. To this end, DMD.Δ48-50 and DMD.Δ45-52 myoblasts were transduced with a dose-range of AdV.Cas9^IN43.IN54^ and, after that, were induced to differentiate. Analogous to previous experiments, parallel transductions with the isogenic vector AdV.Cas9^EX51^ were carried out in DMD.Δ48-50 myoblasts, whereas transduction with the control vector AdV.gRNA^S1^ were done in DMD.Δ48-50 and DMD.Δ45-52 myoblasts. Western blot analysis readily established the presence of Becker-like dystrophins in cultures of differentiated myoblasts DMD.Δ48-50 and DMD.Δ45-52 that had been initially exposed to AdV.Cas9^IN43.IN54^ (Fig. 3c). Consistent with the large sizes of the excised chromosomal DNA fragments, the *de novo* generated dystrophin variants were smaller than those expressed from differentiated wild-type myoblasts (Fig. 3c). Importantly, in line with the *DMD* editing frequencies at the DNA level, the amounts of dystrophin molecules induced by AdV.Cas9^IN43.IN54^ were similar to those achieved by using the isogenic vector AdV.Cas9^EX51^ (Fig. 3c). As expected, parallel transduction experiments in which the control vector AdV.gRNA^S1^ was used, did not lead to detectable amounts of dystrophin (Fig. 3c).

**Figure 4:**
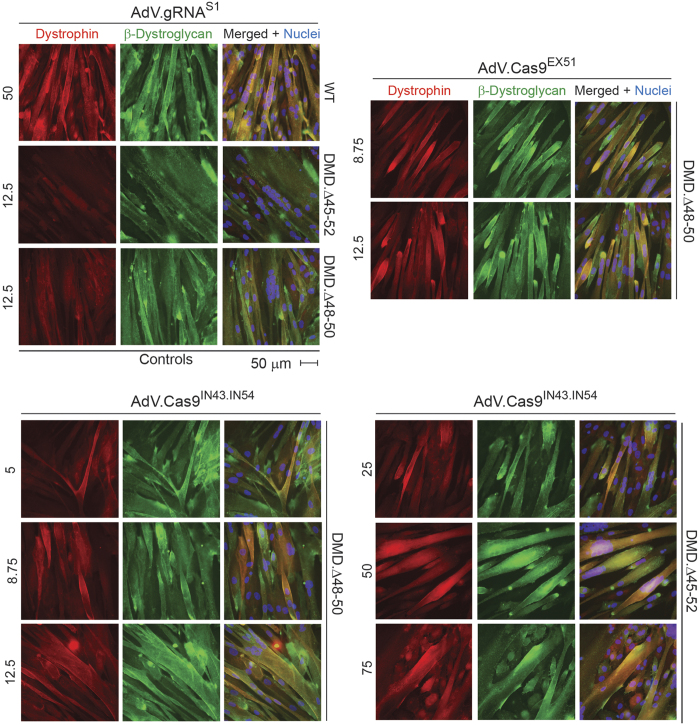
Dystrophin and β-dystroglycan immmunofluorescence microscopy on *DMD*-edited myotubes. Immunostainings for dystrophin and β-dystroglycan were carried out in myotubes differentiated from DMD.Δ48-50 and DMD.Δ45-52 myoblasts transduced with the AdV.Cas9^IN43.IN54^. Each numeral refers to multiplicities of infection (MOI). The same immunostainings were also done in myotubes differentiated from DMD.Δ48-50 myoblasts transduced with AdV.Cas9^EX51^ at the indicated MOI. Myotubes derived from DMD.Δ48-50 and DMD.Δ45-52 transduced with AdV.gRNA^S1^ at an MOI 12.5 IU/cell served as negative controls. Myotubes isolated from a healthy donor (WT) and transduced with AdV.gRNA^S1^ provided for positive controls.

To complement the previous data, myotube cultures parallel to those processed for western blot analysis, were subjected to dual-color immunofluorescence microscopy. This assay permits the simultaneous detection of dystrophin and β-dystroglycan, a key component of the DAPC. Consistent with the western blot results, the immunofluorescence microscopy data confirmed rescue of dystrophin synthesis in DMD.Δ48-50 and DMD.Δ45-52 myotubes whose progenitors had been exposed to AdV.Cas9^IN43.IN54^ or AdV.Cas9^EX51^ (Fig. 4). Furthermore, a degree of AdV.Cas9^IN43.IN54^ dose-dependent increase in dystrophin amounts could be discerned corroborating the data retrieved by qPCR (Fig. 3b). Importantly, co-localization of dystrophin- and β-dystroglycan-specific signals could be detected in *DMD*-edited myotubes (Fig. 4). These data suggest the stabilization and proper assembly of β-dystroglycan at the plasma membrane, likely owing to its linkage to the cysteine-rich domains of the *de novo* synthesized dystrophin molecules.

Taken our data together, we demonstrate that *DMD* editing based on AdVs encoding RGN multiplexes induces robust synthesis of Becker-like dystrophins in unselected muscle cell populations. Moreover, notwithstanding differences specific to each of the experimental settings, namely the use of different gRNAs, our data suggest that AdV delivery of two RGNs can yield *DMD* repair levels similar to those achieved by using AdV transfer of single, mutation-specific, RGNs.

## Discussion

Sequence-specific programmable nucleases have dramatically accelerated progress in genome editing and are opening up alternative therapeutic options for tackling acquired and inherited disorders, including DMD^30^. Yet, advancing the testing and application of *DMD* editing requires, amongst other developments, improved methods for delivering the necessary, and often sizable, molecular tools into target cells. The delivery issue is even more critical in the case of multiplexing strategies since these require simultaneous introduction of different nuclease complexes into target cell nuclei^13^. In this regard, the integration of viral vectors and RGNs, as *bona fide* gene delivery vehicles and versatile programmable nucleases, respectively, has the potential to facilitate and broaden the applicability of multiplexing strategies for *DMD* editing in *ex vivo* and *in vivo* settings^31^.

AdVs constitute appealing delivery systems owing to their large packaging capacity, strict episomal nature and fast kinetics of transgene expression in dividing and post-mitotic target cells^32^,^33^. Indeed, previous work carried out in our laboratory has shown that AdVs yield high-level and transient expression of transcription activator-like effector nucleases and Cas9 nucleases in clinically relevant cell types, including muscle progenitor cells and non-muscle cells with myogenic capacity^20^,^28^,^34^. In the current work, we have demonstrated that “all-in-one” dual RGN-encoding AdVs trigger short- and long-range removal of *DMD* segments encompassing out-of-frame sequences. Both of these gene editing strategies resulted in *DMD* reading frame restoration and ensuing dystrophin detection in target cell populations without the need for implementing expedients to select *DMD*-edited cells or nuclease-exposed cell fractions. Moreover, the frequencies of *DMD* repair achieved by AdV delivery of RGN multiplexes for long-range intragenic deletions were similar to those obtained by AdV transfer of single RGNs for mutation-specific reading frame resetting and DNA-borne exon skipping^20^.

The excision of the large *DMD* segment encompassing exons 44 through 54, shortens the internal rod-shaped portion of dystrophin composed of a series of spectrin-like repeats interrupted by proline-rich segments called hinges^35^. This internal dystrophin portion is, to a certain degree, dispensable as indicated by the fact that the vast majority of the in-frame deletions causing BMD are also located between exon 45 and exon 55 (ref. 36). In this regard, large in-frame *DMD* deletions reducing the length of the rod-shaped portion of dystrophin have been linked to BMD phenotypes that are milder than those caused by certain shorter deletions^37^,^38^,^39^. For instance, it has been shown that extensive in-frame deletions removing hinge 3 (i.e. exons 50–51) correlate with milder BMD phenotypes when compared to smaller deletions that preserve hinge 3^37^. Presumably this is the result of the altered combination of primary amino acid sequences impacting the higher-order structures of dystrophins.

The amenability of RGN multiplexing for inducing exon deletions and thereby rescuing dystrophin synthesis is also supported by a recent cluster of studies involving experiments in the mouse model DMD^*mdx*^ whose mild dystrophic phenotype is caused by a premature stop codon in *Dmd* exon 23 (refs. 22, 23, 24, 25). In particular, intramuscular co-injection of a pair of conventional serotype-5 AdVs encoding Cas9 and gRNAs, resulted in the excision of *Dmd* exon 23 and dystrophin protein synthesis in transduced myofibers^22^. In other studies, systemic and local co-injections of pairs of rAAVs encoding gRNAs and either *S. pyogenes* Cas9 or the smaller orthologue *Staphylococcus aureus* Cas9 (SaCas9), equally led to the excision of *Dmd* exon 23 and restoration of dystrophin synthesis in transduced striated muscles^23^,^24^,^25^. Together, these studies provided proof-of-concepts for *in vivo* muscle-directed gene repair based on viral vector delivery of RGN multiplexes. However, conventional serotype-5 AdVs have a reduced tropism for human muscle progenitor cells and non-muscle cells with myogenic capacity^26^,^27^, while the limited cargo of rAAVs (i.e. ~4.7 kb) restricts the flexibility with which dual RGN-encoding rAAVs can be constructed (e.g. inclusion of sizable constitutive or muscle-specific promoters). In addition, “all-in-one” rAAVs encoding SaCas9 together with gRNA pairs appeared to yield lower frequencies of targeted DNA manipulations when compared to those resulting from rAAVs encoding SaCas9 and gRNA molecules separately^25^.

The repair of defective *DMD* alleles via RGN multiplexing has been further validated in patient-derived myoblasts^19^,^20^ and human iPSCs^21^. In addition, these studies demonstrated that RGN multiplexing can induce large *DMD* excisions, generating internally truncated, but functional, dystrophins^19^,^20^,^21^. However, the very low efficiencies in deleting these large chromosomal segments achieved by using non-viral delivery methods, required the implementation of selection schemes and clonal analysis for the isolation of *DMD*-edited cells^19^,^21^. In contrast, here we report that generating targeted, long-range, *DMD* deletions by “all-in-one” AdV transduction of RGN multiplexes, results in the direct, selection-free, detection of *de novo* dystrophin proteins in bulk target cell populations. The delivery of dual RGN complexes by single AdV particles presumably assures that the various nuclease components are present in target cell nuclei at the same time, in high amounts and at a fixed stoichiometry. The synchronous generation of the paired chromosomal DSBs maximizes the chance that end-to-end ligation of the distal chromosomal termini results in the deletion of the intervening sequence. We showed that dual RGN-encoding AdVs induce short- and long-range genomic deletions correcting over 10% of defective *DMD* alleles.

Studies in severely dystrophic mouse models indicate that dystrophin amounts as low as 4% of the wild-type level confer a clear improvement in survival and motor function^40^,^41^. Hence, if low to moderate levels of dystrophin can equally benefit patients with DMD, therapeutic gene correction will be mostly dependent on delivering in a safe manner gene-edited myogenic cells or gene-editing vector particles to large portions of the striated musculature^31^.

Towards clinical translation, the use of fiber-modified “all-in-one” AdVs encoding single or dual RGNs will permit testing *DMD* editing strategies in human muscle progenitor cells and non-muscle cells with myogenic capacity^42^,^43^, harboring mutations which are not represented in current animal models^44^. These experiments are expected to contribute to assessing intended and unwanted genome-modification events caused by the interaction of specific *DMD* editing reagents with the human genome. The present research should also profit from and build upon the development of sensitive and unbiased assays for genome-wide DSB detection^45^,^46^.

In conclusion, we have demonstrated that AdVs encoding RGN multiplexes designed to remove a single exon or the major *DMD* mutational hotspot offer a flexible and robust NHEJ-mediated *DMD* repair strategy. This research is expected to facilitate the screening and testing of genetic therapies involving the *in situ* repair of defective *DMD* loci in patient-own cells.

## Materials and Methods

### Cells

The human cervix carcinoma HeLa cells (American Type Culture Collection) and the *E1*- and *E2A*-complementing AdV packaging cell line PER.E2A were cultured as previously described^20^,^47^.

The origin of and the cultures conditions for the human myoblasts harboring *DMD* intragenic deletions Δ48-50 or Δ45-52 or the wild-type *DMD* genotype, herein referred to as DMD.Δ48-50, DMD.Δ45-52 and WT, respectively, have also been detailed elsewhere^48^. The various cell batches used in this study were mycoplasma-free.

### gRNAs design and validation

The design and validation of the gRNAs, whose expression is controlled by the human U6 RNA polymerase III promoter, have been described before^20^. The gRNA cassettes gI43, gI52, gI53 and gI54.2 (hereinafter named gIN43, gIN52, gIN53 and gIN54, respectively) target sequences present in *DMD* introns 43, 52, 53, and 54, respectively. The gRNA paired cassettes gIN52/gIN53 and gIN43/gIN54 were employed to trigger short- and long-range intragenic *DMD* excisions, respectively. The individual gRNA cassettes gE51.2 and gE53 (hereinafter named gEX51 and gEX53, respectively), used as references for *DMD* repair based on the delivery of single RGN complexes, target *DMD* sequences located in exon 51 and 53, respectively.

The DNA target sequences of each gRNA are listed in Supplementary Table S1.

### Plasmids

Standard recombinant DNA techniques were applied for the construction of the various AdV transfer constructs and qPCR standard-curve plasmids^49^. To generate the AdV transfer plasmid AU53_pAd.Shu.gI52.gI53.PGK.Cas9.SV40pA, plasmids pLV.gI52 and pLV.gI53 (ref. 20) were digested with *XhoI* (Thermo Scientific) and the resulting fragments, containing the *gI52* and *gI53* expression units, were isolated and ligated in two consecutive steps into the Eco105I and Eco147I restriction sites of pAdSh.PGK.Cas9 (Addgene plasmid #58253) (ref. 28), respectively. Next, the full-length *E1*- and *E2A*-deleted (i.e. second-generation) fiber-modified AdV molecular clone AU57_pAdV^Δ2^gI52.gI53.PGK.Cas9.F^50^ was assembled via homologous recombination (HR) after the transformation of *E. coli* cells BJ5183^pAdEasy-2.50^ (ref. 50) with the MssI-treated AU53_pAd.Shu.gI52.gI53.PGK.Cas9.SV40pA, as detailed elsewhere^50^,^51^. Similarly, AdV transfer plasmid AU04_pAd.Shu.gI54.gI43.PGK.Cas9.SV40pA was obtained by cloning *gI43* and *gI54* expression units from XhoI-treated pLV.gI43 and pLV.gI54.2 (ref. 20) into the Eco147I and Eco105I restriction sites of pAdSh.PGK.Cas9 (Addgene plasmid #58253) (ref. 28), respectively. The resulting AdV transfer plasmid was used for the construction of the *E1*- and *E2A*-deleted fiber-modified AdV molecular clone AU08_pAdV^Δ2^gI54.gI43.PGK.Cas9.F^50^, according to the protocol detailed elsewhere^50^,^51^.

The construction of the AdV molecular clones AU10_pAdV^Δ2^gE53.P.Cas9.F^50^, AU12_pAdV^Δ2^gE51.2.P.Cas9.F^50^ and pAdV^Δ2^P.Cas9.F^50^ used as molecular weight references in the structural analysis of AdV genomes, have been described before refs 20 and 28.

To generate the standard-curve plasmids for the qPCR assays, genomic DNA from cell populations containing RGN-induced *DMD* deletions, were subjected to PCR for amplifying DNA sequences encompassing the *de novo* generated junctions. The primer sequences, the PCR mixture compositions and the cycling parameters are specified in the Supplementary Tables S2 and S3. Agarose gel electrophoresis in 1× TAE buffer was performed for the detection of the resulting PCR products. Next, PCR amplicons were purified using the JETquick Gel Extraction Spin kit (Genomed), according to the manufacturer’s recommendations. Subsequently, purified PCR products were cloned into the pJET1.2/blunt backbone using the CloneJET PCR cloning Kit (Thermo Scientific), according the manufacturer’s instructions. These maneuvers gave rise to plasmids AV31_jDEL.I52-I53 and AV24_jDEL.I43-I54. The structural integrity of the generated plasmids was assessed by restriction fragment length analysis. The nucleotide sequences of the inserts were confirmed by Sanger sequencing (BaseClear, Leiden, the Netherlands). Similarly, conventional recombinant DNA techniques were applied to generate AL05_pDMD. This construct, containing DNA sequences from *DMD* intron 43, was used for generating standard curves for the quantification of a control genomic region via qPCR assays. The complete DNA sequence of AL05_pDMD is presented in Supplementary Table S4.

### Production and characterization of Adenoviral vectors

The AdV molecular clones AU57_pAdV^Δ2^gI52.gI53.PGK.Cas9.F^50^ and AU08_pAdV^Δ2^gI54.gI43.PGK.Cas9.F^50^ were used for the production of the fiber-modified *E1*- and *E2A-* deleted AdVs AdV.Cas9^IN52.IN53^ and AdV.Cas9^IN43.IN54^, respectively. The procedures used to generate, purify and titrate these AdVs have been described in detail before^28^,^51^. The generation and characterization of the fiber-modified *E1*- and *E2A*-deleted AdVs AdV^Δ2^P.Cas9.F^50^, AdV^Δ2^P.Cas9.gE53.F^50^, AdV^Δ2^P.Cas9.gE51.2.F^50^ (herein referred as AdV.Cas9, AdV.Cas9^EX53^, AdV.Cas9^EX51^, respectively), as well as the negative control vector AdV ^Δ2^U6.gRNA^S1^.F^50^ (herein named AdV.gRNA^S1^) have been described elsewhere^20^,^28^. The titers of the purified AdV stocks expressed in genome copies per ml (GC/ml) and infectious units per ml (IU/ml) together with their ratios are listed in Supplementary Table S5. The former and latter titers were determined by using the QuantiT PicoGreen dsDNA Assay Kit (Invitrogen) and TCID_50_ assays, respectively, as described elsewhere^50^.

The structural integrity of packaged AdV genomes was assessed by restriction fragment length analysis (i.e. Kpn2I and BglII) of DNA isolated from the respective purified particles as described before^28^,^33^,^51^.

### Transduction experiments

Gene editing experiments were initiated by seeding DMD.Δ45-52 and DMD.Δ48-50 myoblasts at a density of 3×10^4^ cells per well of 24-well plates (Greiner Bio-One) pre-coated with a 0.1% (w/v) gelatin solution (Sigma-Aldrich). The next day, DMD.Δ45-52 myoblasts were exposed in 500-μl volumes to different MOIs of AdV.Cas9^IN52.IN53^, whereas DMD.Δ45-52 and DMD.Δ48-50 myoblasts were exposed to different MOIs of AdV.Cas9^IN43.IN54^. DMD.Δ48-50 myoblasts transduced with AdV.Cas9^EX51^ served as a reference for *DMD* reading frame repair by a gene editing approach based on the delivery of a single RGN complex. Negative controls were provided by mock-transduced DMD.Δ45-52 and DMD.Δ48-50 myoblasts. Three days post-transduction, the inocula were discarded and the cells were transferred into cell culture vessels containing regular growth medium. Seven days post-transduction, genomic DNA was isolated and the intragenic *DMD* deletion frequencies were determined by qPCR assays as indicated below. The frequencies of targeted DSB formation in AdV.Cas9^EX51^-transduced DMD.Δ48-50 myoblasts were assessed by TIDE analysis as indicated elsewhere^20^,^52^.

### qPCR assays

The frequencies of *DMD* intragenic deletions were assessed by qPCR assays. To this end, total cellular DNA from mock- or AdV-transduced cells was extracted at 7 days post-transduction by using the DNeasy Blood & Tissue Kit (Qiagen) according to the manufacturer’s instructions. Next, 5 ng of genomic DNA were diluted in 20-μl reaction mixtures with 1 × iQ^TM^ SYBR Green Supermix (Bio-Rad) and 150 nM of each primer. The primers were designed with the aid of the open source Primer3 program (http://frodo.wi.mit.edu/) and their sequences are specified in the Supplementary Table S6. All samples were amplified in triplicate. Parallel qPCR amplifications targeting DNA sequences located in intron 43 of the *DMD* locus provided for internal controls. The amplifications were carried in a CFX Connect^TM^ Real-Time System (Bio-Rad), according to the thermal cycling protocols indicated in Supplementary Table S7. After each run, the baseline thresholds and the estimated copy numbers were auto-calculated by Bio-Rad CFX Manager 3.1. The copy numbers of genomic short- and long-range *DMD* deletions were determined on the basis of standard curves using serial dilutions (ranging from 2.26 × 10 to 2.32 × 10^4^ copies) of AV31_jDEL.I52-I53 and AV24_jDEL.I43-I54 plasmids, respectively. For the internal control samples, standard curves were made on the basis of serial dilutions (ranging from 7.56 × 10 through 7.56 × 10^4^ copies) of reference plasmid AL05_pDMD. Finally in order to estimate deletion frequencies, the copy numbers of genomic deletions were normalized, on a per-sample basis, for the copy numbers of the internal control. The data were plotted with the aid of Microsoft Office Excel 2010.

### Qualitative detection of long-range *DMD* deletions

Genomic DNA extracted from mock- or AdV-transduced cells was subjected to PCR assays for amplifying DNA fragments spanning the expected DNA junctions formed after the coordinated action of RGN pairs. The primer sequences, PCR mixture compositions and cycling parameters are listed in the Supplementary Tables S2 and S3. Agarose gel electrophoresis in 1× TAE buffer was performed for the detection of the resulting PCR products.

### Western blot analysis

The DMD.Δ45-52 and DMD.Δ48-50 myoblasts were first seeded and transduced as indicated above (Transduction experiments). Next, DMD.Δ45-52 myoblasts were exposed to different MOIs of AdV.Cas9^IN52.IN53^, whereas DMD.Δ45-52 and DMD.Δ48-50 myoblasts were exposed to different MOIs of AdV.Cas9^IN43.IN54^. References were provided by parallel transductions of DMD.Δ45-52 and DMD.Δ48-50 myoblasts with a dose-range of AdV.Cas9^EX53^ and AdV.Cas9^EX51^, respectively. Cultures containing myotubes derived from mock-transduced wild-type myoblasts served as positive controls. Negative controls were provided by exposing DMD.Δ45-52 and DMD.Δ48-50 cells at an MOI of 12.5 IU/cell to control vector AdV.gRNA^S1^.

Three days post-transduction, the various inocula were replaced by mitogen-poor differentiation medium to trigger myoblast differentiation and fusion. The differentiation medium consisted of phenol red-free DMEM supplemented with 0.05% bovine serum albumin (Sigma-Aldrich), 10 ng/ml epidermal growth factor (Sigma-Aldrich), 1 mM creatine monohydrate (Sigma-Aldrich), 50 μg/ml uridine (Sigma-Aldrich), 100 ng/ml pyruvic acid (Sigma-Aldrich), 1× Penicillin/Streptomycin (Life Technologies) and 1× GlutaMax (Life Technologies). After 4 to 5 days in differentiation medium, the myotube-containing cultures were lysed by incubation on ice for 30 min in 50 μl of RIPA buffer (Thermo Scientific) supplemented with a protease inhibitor cocktail (cOmplete^TM^ Mini, Sigma-Aldrich). Next, the cell lysates were diluted in 4× sample buffer and 20× reducing agent (both from Bio-Rad) and incubated at 95 °C for 5 min. Protein samples and 15 μl of HiMark^TM^ Prestained Protein Standard (Thermo Scientific) were loaded in a 3–8% Criterion^TM^ XT Tris-Acetate precast gel (Bio-Rad). The gel was placed in an ice-cooled Criterion^TM^ Cell and was run in XT Tricine running buffer (both from Bio-Rad) first for 1 h at 75 V (0.07 A) and then for 2.5 h at 150 V (0.12 A). Subsequently, the resolved proteins were transferred with the aid of a Trans-Blot^®^ Turbo^TM^ Midi PVDF pack and a Trans-Blot Turbo^TM^ system (both from Bio-Rad), according to the manufacturer’s recommendations for high molecular-weight proteins (2.5 A, 25 V, 10 min). The PVDF membrane was blocked overnight in TBS with 0,05% (v/v) Tween 20 (TBST, Thermo Scientific) supplemented with 5% (w/v) Elk milk (Campina) and subsequently was washed in TBST. Next, the membrane was incubated with a rabbit polyclonal antibody directed against dystrophin (ab15277; abcam) or against α/β Tubulin (CST #2148) diluted in TBST with 5% (w/v) Elk milk. After an overnight incubation period at 4 °C, the membrane was washed in TBST and incubated for 4 h at 4 °C with an anti-rabbit IgG secondary antibody conjugated to horseradish peroxidase (IgG-HRP; Santa Cruz) diluted in TBST. Proteins were detected by using horseradish peroxidase substrate Pierce^TM^ ECL2 (Thermo Scientific) following the manufacturer’s specifications with the aid of Super RX-N X-Ray film (Fujifilm).

The schematic representation of the Becker-like dystrophin molecules and their respective molecular weights resulting from AdV-based *DMD* editing procedures involving short- and long-range deletions were assembled with the aid of the eDystrophin website (http://edystrophin.genouest.org/) (ref. 53).

### Immunofluorescence microscopy

DMD.Δ45-52 and DMD.Δ48-50 myoblasts were seeded and transduced with AdV.Cas9^IN43.IN54^ as indicated above (Transduction experiments). Cultures of differentiated wild-type myoblasts that had been transduced with control vector AdV.gRNA^S1^ at an MOI of 50 IU/cell served as positive controls for dystrophin immunodetection assays, whereas exposing DMD.Δ45-52 and DMD.Δ48-50 myoblasts to the same vector at an MOI of 12.5 IU/cell provided for negative controls. Cultures of differentiated DMD.Δ48-50 myoblasts that had been transduced with different MOIs of AdV.Cas9^EX51^ served as additional reference samples.

Three days post-transduction, myoblast differentiation and fusion were triggered by adding mitogen-poor medium, as indicated above (western blot analysis). The myotube-containing cultures of myoblasts with wild-type, DMD.Δ45-52 and DMD.Δ48-50 genotypes were stained as described elsewhere^20^ after 3, 4 and 5 days in differentiation medium, respectively. The applied primary antibodies were a rabbit polyclonal antibody directed against the C-terminus of dystrophin (ab15277; abcam) and the NCL-b-DG mouse monoclonal antibody specific for β-dystroglycan (clone 43DAG1/8D5; Novocastra).

## Additional Information

**How to cite this article**: Maggio, I. *et al.* Adenoviral vectors encoding CRISPR/Cas9 multiplexes rescue dystrophin synthesis in unselected populations of DMD muscle cells. *Sci. Rep.* **6**, 37051; doi: 10.1038/srep37051 (2016).

**Publisher’s note:** Springer Nature remains neutral with regard to jurisdictional claims in published maps and institutional affiliations.

## Supplementary Material

Supplementary Information
