# Supplementary material for: Adenoviral vectors encoding CRISPR/Cas9 multiplexes rescue dystrophin synthesis in unselected populations of DMD muscle cells
Source: Sci Rep. 2016 Nov 15;6:37051. doi: 10.1038/srep37051 (PMC5109245; doi:10.1038/srep37051)
Supplement: Supplementary Information [file srep37051-s1.pdf]

Adenoviral vectors encoding CRISPR/Cas9 multiplexes rescue dystrophin synthesis in unselected populations of DMD muscle cells

Ignazio Maggio, Jin Liu, Josephine M. Janssen, Xiaoyu Chen and Manuel A.F.V. Gonçalves

SUPPLEMENTARY FIGURES

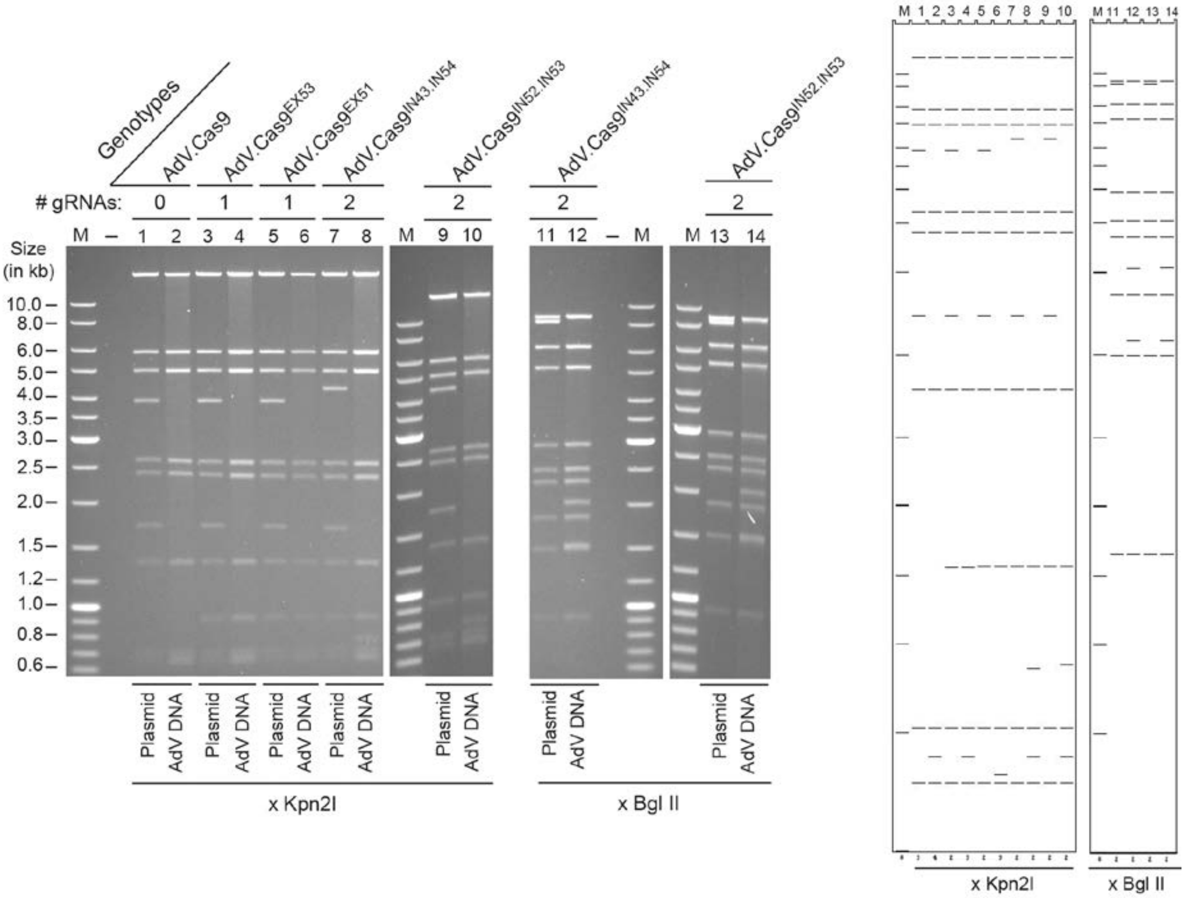

Supplementary Fig. S1 Structural analysis of AdV genomes encoding RGN components. Restriction fragment length analysis was performed on recombinant genomes isolated from CsCl-purified AdV particles. Left panel, in-gel restriction enzyme patterns resulting from exposing the indicated AdV genomes to Kpn2I or BglII. The parental AdV molecular clones (Plasmid), digested with the same restriction enzymes, provided for additional DNA molecular weight references. Right panel, predicted (*in silico*) restriction enzyme patterns generated with the aid of the Gene Construction Kit software package (Version 4.0). Lanes M, GeneRuler DNA ladder mix (Fermentas).

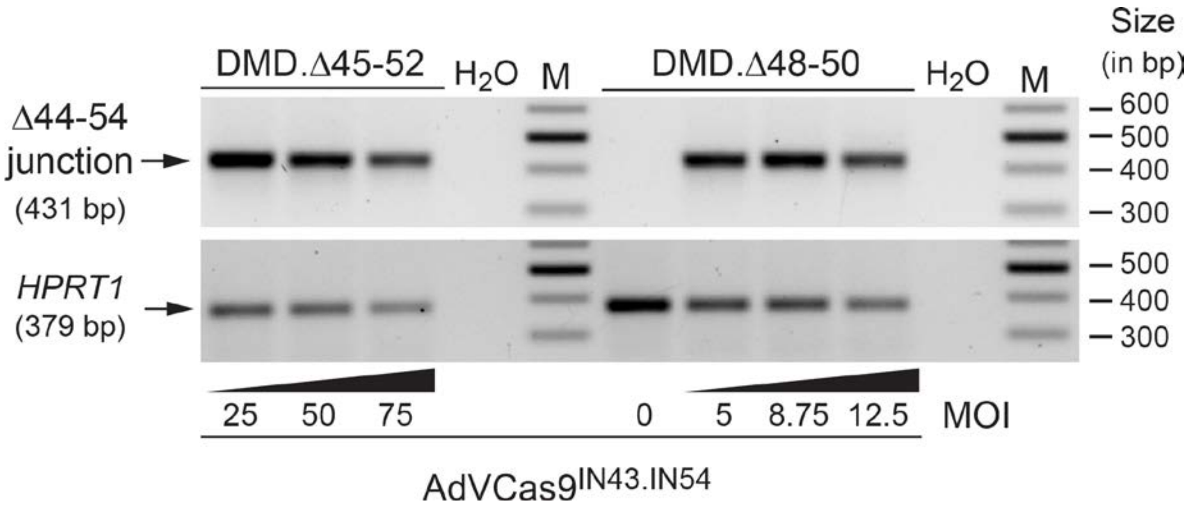

Supplementary Fig. S2 PCR detection of major *DMD* mutational hotspot excisions in myoblasts transduced with AdV.Cas9$^{IN43.IN54}$. DMD.Δ45-52 and DMD.Δ48-50 myoblasts were transduced with AdV.Cas9$^{IN43.IN54}$ at the indicated multiplicities of infection (MOI). At 7 days post-transduction, genomic DNA was isolated and PCR assays were carried out with primer pairs flanking the RGN-induced intronic *DMD* junction. Negative controls were provided by parallel PCR amplifications on nuclease-free water and genomic DNA templates isolated from mock-transduced DMD.Δ48-50 myoblasts. Arrows mark the sizes and positions of amplicons diagnostic for the long-range multi-exon deletion (Δ44-54 junction) and the internal control *HPRT1* target sequences. Lanes M, GeneRuler DNA Ladder Mix (Fermentas).

Adenoviral vectors encoding CRISPR/Cas9 multiplexes rescue dystrophin synthesis in unselected populations of DMD muscle cells

Ignazio Maggio, Jin Liu, Josephine M. Janssen, Xiaoyu Chen and Manuel A.F.V. Gonçalves

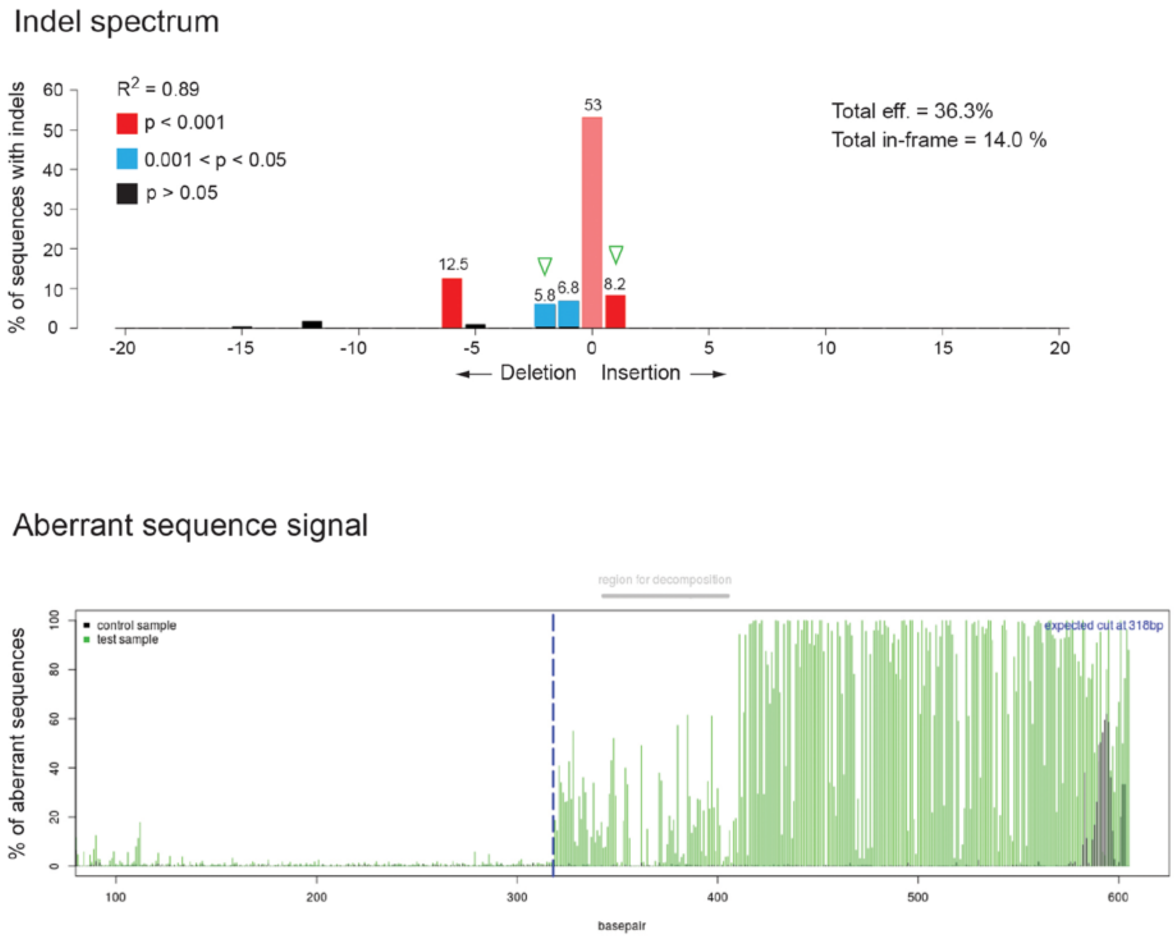

Supplementary Fig. S3 Tracking of Indels by DEcomposition (TIDE) analysis in DMD.Δ48-50 myoblasts transduced with AdV.Cas9^{EX51}. DMD.Δ48-50 myoblasts were transduced with 12.5 IU/cell of AdV.Cas9^{EX51}. TIDE analysis was performed at 7 days post-transduction by deploying *DMD* exon 51 capillary sequencing trace data retrieved from AdV- and mock-transduced cells. Upper panel, indel spectrum detected in DMD.Δ48-50 myoblasts transduced with AdV.Cas9^{EX51}. Vertical open arrowheads indicate the indel types yielding restored *DMD* reading frames by NHEJ-mediated repair of targeted DSBs. Lower panel, Aberrant sequence signal caused by targeted DSB-derived indels generated in the treated sample (green) compared to the sequence signal corresponding to the control, mock-transduced, cells (black). The vertical dotted line indicates the expected break site; the horizontal grey line indicates the region used for decomposition analysis.

Adenoviral vectors encoding CRISPR/Cas9 multiplexes rescue dystrophin synthesis in unselected populations of DMD muscle cells

Ignazio Maggio, Jin Liu, Josephine M. Janssen, Xiaoyu Chen and Manuel A.F.V. Gonçalves

SUPPLEMENTARY TABLES

Supplementary Table S1
Target site sequences of gRNAs targeting *DMD* locus

gRNA	Target site sequence with PAM (5' → 3')	Target site region
gIN52	GATGGCAAAGCAAACTCCTGTGG	Intron 52
gIN53	GTAATCTGGATATAGTTGGCTGG	Intron 53
gIN43	GTTACATACAGGCTAGGGAGTGG	Intron 43
gIN54	GGTACACAATAGGTACGGAATGG	Intron 54
gEX51	ACCAGAGTAACAGTCTGAGTAGG	Exon 51
gEX53	GAAAGAATTCAGAATCAGTGGG	Exon 53

Supplementary Table S2

Primer pairs and composition of PCR mixtures.

Target	Primer code	Primers (5' → 3')	dNTPs	$MgCl_2$	GoTaq Flexi Buffer	GoTaq	Amplicon size
Single-exon deletion junction	# 1084	GCATCAAGGGAGCTAGTGTAAC (0.2 µM)	0,4 mM	2 mM	1x Colorless	2 U	358 bp
	# 1080	AGAGAATGGGGGGAGAAAGTG (0.2 µM)					
Multi-exon deletion junction	#1052	GGTAGTAAGTCAGATGTGATG (0.2 µM)	0.4 mM	2 mM	1x Colorless	2 U	431 bp
	#1109	GAATGGCAGATCTAATGTACAG (0.2 µM)					
Target site gEX51	#1097	GCATGAGAATGAGCAAAATCG (0.2 µM)	0.4 mM	2 mM	1× Colorless	2 U	622 bp
	#1099	CTGAGAGAGAAACAGTTGCC (0.2 µM)					
Target site *HPRT1*	#1005	TCACTGTATTGCCCAGGTTGGTG (0.2 µM)	0.4 mM	3 mM	1x Colorless	2 U	379 bp
	#1006	GAAAGCAAGTATGGTTTGCAGAGAT (0.2 µM)					

Note: GoTaq® G2 Flexi DNA Polymerase (Promega) were used for the PCR amplifications.

Supplementary Table S3
PCR cycling parameters

Target	Initial denaturation	Denaturation	Annealing	Elongation	# cycles	Final elongation
Single-exon deletion junction	95 °C	95 °C	67 °C (decrease 0.5 °C/cycle)	72 °C	10	
	5 min	25 sec	25 sec	25 sec		
		95 °C	62 °C	72 °C	30	72 °C
		25 sec	25 sec	25 sec		3 min
Multi-exon deletion junction	95 °C	95 °C	66 °C (decrease 0.5 °C/cycle)	72 °C	10	
	5 min	25 sec	25 sec	25 sec		
		95 °C	61 °C	72 °C	30	72 °C
		25 sec	25 sec	25 sec		3 min
Target site gEX51	95 °C	95 °C	62 °C (decrease 0.5 °C/cycle)	72 °C	10	
	3 min	30 sec	30 sec	30 sec		
		95 °C	57 °C	72 °C	25	72 °C
		30 sec	30 sec	30 sec		3 min
Target site *HPRT1*	95 °C	95 °C	63 °C (decrease 5.5 °C/cycle)	72 °C	10	
	3 min	25 sec	25 sec	25 sec		
		95 °C	58 °C	72 °C	25	72 °C
		25 sec	25 sec	25 sec		2 min

Supplementary Table S4

Sequence of plasmid AL05_pDMD used to set-up the standard curves for the internal control of the qPCR assay

CTAAATTGTAAGCGTTAATATTTTGTTAAAATTCGCGTTAAATTTTTGTTAAATCAGCTCATTTTTTAACCAATAGGCCGAAATCGGCAAAATC
CCTTATAAATCAAAAGAATAGACCGAGATAGGGTTGAGTGGCCGCTACAGGGCGCTCCCATTCGCCATTCAGGCTGCGCAACTGTTGGGAAG
GGCGTTTCGGTGCGGGCCTCTTCGCTATTACGCCAGCTGGCGAAAGGGGGATGTGCTGCAAGGCGATTAAGTTGGGTAACGCCAGGGTTTTC
CCAGTCACGACGTTGTAAAACGACGGCCAGTGAGCGCGACGTAATACGACTCACTATAGGGCGAATTGGCGGAAGGCCGTCAAGGCCTAGG
CCATAAGGTAAAGAGGACACCAAATACAGTAAATAATAACATAATAATAAAGAAACCTTATGAATTCTATTCAGTGATATCCATATTGAGGT
ATTTAAGGTGAATGATACTGATGTCAGCAGTTTACCTGGATATGCATCAAAAATATGATTGATTGATGGATGGATCGGAGGAAGGAAAAGAT
ATGTGATCAAAACGAGTATAGTAAAATGTTCATGGTGGAATCTAGATATATAGATATCACTTGTAATTTTTAAAAATGTTATTGCATTTTTGA
AATTTGTTATGTTAAAATATTGGGTGAAAATTCTGATGCCCTGGCCATGTCCCAAATCAGTTGAATCAGATCTCTGGTGTATTTTTTTTTTAAG
ATCCTTGGATGACTCCATTGTACAGCAAGTTTGCAAACCACTATTCTAACTTCTCACCACCCCCTTCTTTATAGCTTACAAAATTATATTTTTA
AATGTCAAATGTCACAATTTCACTTTCCTTCTTCAAGAACTGATTAGACTTCTAGCTTCTCCTATGCTCTCATCTTGCATAACGTTCATTTGCAC
GTGGTTAAAACTGATAGGCAATAAATAATAACCCATCTGTCTTTTCCTGACCCAACATAACTAAAACCTTCCTTTCAAGGCGAAAATGAACTA
TAATCTCACAGACAAGAGTAGCTATAGACAACTAATTCTTTCTGGTCTCTCGTGGTCATGGGACTATTCACCACTGCCGTCCCTGCTGTCTATT
TGGTGGACCAGCCAGTTTTACTGTGACCCATACCATGGCTCTTGAGAAGCATGAAGCTGCCGAAATCGCAGTTGTAGGGTTAATGGAAAACA
TGGTGAAGTATAGCTTACATATATATACAATTTTGGCACAAAATGAAGCCTAAGAAATTGTACCGGGCTGGGTGTGGTGACTCATGCCTGTAA
TCCCAGCACTTTGAGAGACCAAGGTGGGCGGATCACCTGAGGTCGGGAGTTCGAGACCAGACTGAGCAACAT
GGAGAAACCCTCTCTACTTAAAATACAAAATTAGCCAGGCATGGTGGCGCATGCCTGTAACCCCAGCTACTCAGGAGGCTAAGGCAAGAGA
ATCTCTTCCACCCTGGAGGCATAGGTTGTGGTGAGAGAAGATCACACCATTGCACTCCAGCTGGGGCAACAAGAGCGAAGCTCCGTCTCAAT
AAATAAATAAATAAATAAATAAATAAATAAATAAATAAATAAATAAAAGAAAAAGAAATTGTACCAGGAAGTGGATTGGGAATGCTCTATA
TATTCTCCCCCTCCAAGAAGTTTTTTTAATTTATTGTATTGTGAAAGATCTGCAGCTATTTATTTTGTGTGGTACTCTTTTGCAATAAACTACTT
CTGCATAAATGAACTCAAAAACTTTGATATTCATGAACCTTCATAATGTTGATCTAGTACATCTTGCCCAACTAGCCCTTGGGCCATTCCTTCC
ATGCCACTGGTTGAAAAATATGCTCCCCACCCCAGTAACATCAGCATCAGACCTCTGCAACCAGAAAGATTCCATAGGTAATGAAATCCAAA
TGCTGGCATTTGGAAGTCAAACCAGTGTATTTTGAAATGGAAAGGCAAGGGGAAGTGCATGGAGCAAGTACAGCATGTGCTACAACTTCAGT
TCATACCCTCCTTAAAATACTGTGCAACTAAACTGTCAATTGCCCATTGATAGAGATAGTATCTTTTTCAGCTTTACACCTTCCGAATTCAACA
CACTGCTAGCCTCAGGACTAAGCGCTCAATAAATGTTTATCAAAAGATTGAGTAAATGAATCAGTGGTGAAATAAAATGTACAAACACATAC
GCTTCATACACGTTTAGGAAGCATTTCTAGTGAGCTTTGTGGATATTTTATTTCTTAAAGGTTCTTTTTCTTTCCATGTTCATGTTATTCTTTATT
TTTTAAAATGTTGCTTCTGCTTATTTCTCTTTCTACGGCAGCTATATTTACTTGCCTGAGAGCACAGCATATACTGATGTTAATCAAACTTTAGA
TGAAACACCTAAAATGATAGAAAAGAATCTGCTCTAATTAAATAATAAAGCAACCCCTACAGGTTTAGACATGTGCCTTCCGGTGTGGGAAA
GAAAAAATTTAATGAATGTAGTAGTTTTATGCCAAGAACATTTCCCTCGTGCACCTGTGTTTTAATAAGATAGAATATAAAATAGCAAAAGG
GGCCCGACTTTTGTGATGATATTTACTCATAAGGTAGTAAGTCAGATGTGATGACATTTTCTTTGACTTAGAGCTGCTATACTTGGGTCAGATT
TCAGTTCAGTAAATTTGCAGTGAAGTTGTCTTTCTAACATGGTGTCATCCTGGAACTGCCCTGCTCCCACAGTAAGCTTCCACGGGGTTGGGG
TTGCGCCTTTTCCAAGGCAGCCCTGGGTTTGCGCAGGGACGCGGCTGCTCTGGGCGTGGTTCCGGGAAACGCAGCGGCGCCGACCCTGGGTC
TCGCACATTCTTCACGTCCGTTCGCAGCGTCACCCGGATCTTCGCCGCTACCCTTGTGGGCCCCCCGGCGACGCTTCCTGCTCCGCCCCTAAGT
CGGGAAGGTTCCTTGCGGTTCGCGGCGTGCCGGACGTGACAAACGGAAGCCGCACGTCTCACTAGTACCCTCGCAGACGGACAGCGCCAGGG
AGCAATGGCAGCGCGCCGACCGCGATGGGCTGTGGCCAATAGCGGCTGCTCAGCAGGGCGCGCCGAGAGCAGCGGCCGGGAAGGGGCGGTG

```
CGGGAGGCGGGGTGTGGGGCGGTAGTGTGGGCCCTGTTCCTGCCCGCGCGGTGTTCCGCATTCTGCAAGCCTCCGGAGCGCACGTCGGCAGT
CGGCTCCCTCGTTGACCGAATCACCGACCTCTCTCCCCACACGACTCTAGAGGATCCCCGGGTACCGGTCGCCACCATGGTGAGCAAGGGCG
AGGAGCTGTTCACCGGGGTGGTGCCCATCCTGGTCGAGCTGGACGGCGACGTAAACGGCCACAAGTTCAGCGTGTCCGGCGAGGGCGAGGG
CGATGCCACCTACGGCAAGCTGACCCTGAAGTTCATCTGCACCACCGGCAAGCTGCCCGTGCCCTGGCCCACCCTCGTGACCACCCTGACCTA
CGGCGTGCAGTGCTTCAGCCGCTACCCCGACCACATGAAGCAGCACGACTTCTTCAAGTCCGCCATGCCCGAAGGCTACGTCCAGGAGCGCA
CCATCTTCTTCAAGGACGACGGCAACTACAAGACCCGCGCCGAGGTGAAGTTCGAGGGCGACACCCTGGTGAACCGCATCGAGCTGAAGGG
CATCGACTTCAAGGAGGACGGCAACATCCTGGGGCACAAGCTGGAGTACAACTACAACAGCCACAACGTCTATATCATGGCCGACAAGCAG
AAGAACGGCATCAAGGTGAACTTCAAGATCCGCCACAACATCGAGGACGGCAGCGTGCAGCTCGCCGACCACTACCAGCAGAACACCCCCA
TCGGCGACGGCCCCGTGCTGCTGCCCGACAACCACTACCTGAGCACCCAGTCCGCCCTGAGCAAAGACCCCAACGAGAAGCGCGATCACATG
GTCCTGCTGGAGTTCGTGACCGCCGCCGGGATCACTCTCGGCATGGACGAGCTGTACAAGTAAAGCGGCCGCGACTCTAGAATTTTAAGCTG
TGCCTTCTAGTTGCCAGCCATCTGTTGTTTGCCCCTCCCCCGTGCCTTCCTTGACCCTGGAAGGTGCCACTCCCACTGTCCTTTCCTAATAAAAT
GAGGAAATTGCATCGCATTGTCTGAGTAGGTGTCATTCTATTCTGGGGGGTGGGGTGGGGCAGGACAGCAAGGGGGAGGATTGGGAAGACA
ATAGCAGGCATGCACGTGTACAGGCTAGGGAGTGGGTAGGAGTGGGGGTGAAATCCTCTTAATGTTTATGGTGTCAGTAGATTCAAACTAAA
ATTAGCCTTACAGCCATACTCCTAATAAGGGGCCCCTGGCATATTTAATTGATTTAACAAATTTATCAAAAATAGATAAACTGAAATCTGCCT
TGAAATTAATTACTGTATCTCTATTTTTATAAGAAAATATTTTGGACCCGTTCCCTCTGCCTTATGGGTGCAACTCCCGGCAGAATAGGTCATG
TCTCCTGAAAATAGTTCTTATTTTCTTTACTACTTATGACCTCTTATAGCCTAGAAGTTTTTCCTTGCTATTTCTAGTTGAAATGCTAATCTGGC
ATAATTTCTAGTTGAAATGCTAATCTGGCATAATTTCAGAACTAATTTTCTGTTAATGCCACTTGGAACATCTAAATTCCTCCTTTTTCAATAA
TACTATATTTGTGTGTTGCAAACACACACAAATCATACTATATTTGTGTGTATATATAGTAAATTCCAACTTATAAAAGTAAAATTGGAATTTT
TTTCTTTCTTTGGAAAATTATTTTATAATTGAGAATTATAAATGTCACTTTTTTAAATGCTGCAACCTTTGAGATTGGTTTCAATAAAGTAAAA
CTTAGTAAACATTAAGAAAAATGATAGCTTGATATGTTCACTAATATGGTAAATGAAAACTTTTATGTGTGTATAGGCTCTTGTTAATTATAA
CTACCTTCACAGGAAAAACAGTCTTGTGGAAGGTAATGGTGCCAGTGAGAGAAAATAGAGAAAGTAGAATAGTAGTAAGAGAGAAAATAGA
ATTTCCCGTTTAGAATAAAGCTAAGTAAAAAGTGAATTGTTTCAGTTTTATTTATGTATTTGTAAGTGTGGTCTCATGCCATATATATTCAAAG
ATAGACAGAGATCTTAATCTTTAATTTTTTATGGCCAAAGAAAATGAGTCCCATGTAAAAGGGACATTTTCGTAAGCTGATCTCAAGTGGTGT
ATTTTGATTTGCCGCAGCAGCCTATGGAAGAAATATCATTTTGGCTGCCTAAGAAGAAAAATGTCATACCTTTCTATTTTTTATTCATACTCCG
CATATTTGGAATTCGTATTCTCACCATACTTTTGGGAAGATAACACGAAATTGCCTTACTTGCCTCGAAATCAAAATCTGCCATTCGTTTTAAA
ATAAAATGGCTTTTTCATCTCATTTTCCTTTAGCAGAAAAAAGTAAGTTAAATTTTTTTGTAGCTGTGTTAAGTGTACTGTTCCTCCCTCGTTGA
GAACGAAACAAAGACATATGATTCCATTTACTGTAATTGTTTTGGTTTGCCCAATTACAATAGCAGTAAAATCATTTACACATAGTAAAATGT
TTGAGCCTTAAAGGGCAAGAAGACTAAATTTAGCTGAAGATATACAACTTTCATGTGCCAATGAGGAAATGTATAAAATCAGTTATACATAT
TTTCCTCATTCATAGATATTGAAAATATTCATATATGTGTACATGTATTTGATAACTTAATATTTTCTAAATAACAGAAATTATATACTGTATA
TATATTTGGATATTATGATTATTGTTATTGCCACCATTGAGCACTTACTATGGGCTCAGAAAACTTCACTGTATTATTTTTAATTCTTATAACAA
CCCTGTGAAATATATATTATGCCTATTTCACAAATGAGAACTCTGAAATCAGGAAATTAAATCACTTGCTGAAGTTTACAAAGCACTGTTAGG
CAATAAAACAGAATTTCAAACTCACGTTTGCTTGTCTTCAAATTTTGTATGCTGTACAACCTGATTGTTTTACTACTTGTTCTTAATTTCAAAA
AAAATCTTCATACTAAAAGATGATACTTTGGGAGTTCTAAAGAACATGTTTTTGGCCGGGCGCGGTGGCTCACGCCTGTAATCCCAGCACTTT
GGGAGGCCGAGGCAGAAGGATCACGAGGTCAGGAGATCGAGACCGTCCTGGCCAACAAGGTGAAACCCCGTCTCTACTAAAAAATACAAAA
AATTAGCCGGGCGTGGTGGCGGGCGCCTGTAGTCCCAGCTACTCGGGAAGCTGAGGCGGGAGAAGGCGTGAACCTGGGAGGCAGAGCTTG
TAGTGAGCCGAGATCGCGCCGCTGCACTCCAGCCTGGTTGACAGAGCGAGACTCCGTCTCAAAAATAACAAAACAAAACAAAACAAAACAA
AAAAAACCATGTTTCTTTTCAGAAGGTATACTCATTTGAAGTGGATACCAATTATTTGTATTAAAATTACTTATGGATAAATTTGAATCTGCAA
AAATTAAGTGCAACATTATTTTTTGGCACCTGTTATAGGAGGGTAACTCGAGGGTACCTCTTAATTAACTGGCCTCATGGGCCTTCCGCTCACT
```

```
GCCCGCTTTCCAGTCGGGAAACCTGTCGTGCCAGCTGCATTAACATGGTCATAGCTGTTTCCTTGCGTATTGGGCGCTCTCCGCTTCCTCGCTC
ACTGACTCGCTGCGCTCGGTCGTTCGGGTAAAGCCTGGGGTGCCTAATGAGCAAAAGGCCAGCAAAAGGCCAGGAACCGTAAAAAGGCCGC
GTTGCTGGCGTTTTTCCATAGGCTCCGCCCCCCTGACGAGCATCACAAAAATCGACGCTCAAGTCAGAGGTGGCGAAACCCGACAGGACTAT
AAAGATACCAGGCGTTTCCCCCTGGAAGCTCCCTCGTGCGCTCTCCTGTTCCGACCCTGCCGCTTACCGGATACCTGTCCGCCTTTCTCCCTTC
GGGAAGCGTGGCGCTTTCTCATAGCTCACGCTGTAGGTATCTCAGTTCGGTGTAGGTCGTTCGCTCCAAGCTGGGCTGTGTGCACGAACCCCC
CGTTCAGCCCGACCGCTGCGCCTTATCCGGTAACTATCGTCTTGAGTCCAACCCGGTAAGACACGACTTATCGCCACTGGCAGCAGCCACTGG
TAACAGGATTAGCAGAGCGAGGTATGTAGGCGGTGCTACAGAGTTCTTGAAGTGGTGGCCTAACTACGGCTACACTAGAAGAACAGTATTTG
GTATCTGCGCTCTGCTGAAGCCAGTTACCTTCGGAAAAAGAGTTGGTAGCTCTTGATCCGGCAAACAAACCACCGCTGGTAGCGGTGGTTTTT
TTGTTTGCAAGCAGCAGATTACGCGCAGAAAAAAAGGATCTCAAGAAGATCCTTTGATCTTTTCTACGGGGTCTGACGCTCAGTGGAACGAA
AACTCACGTTAAGGGATTTTGGTCATGAGATTATCAAAAAGGATCTTCACCTAGATCCTTTTAAATTAAAAATGAAGTTTTAAATCAATCTAA
AGTATATATGAGTAAACTTGGTCTGACAGTTACCAATGCTTAATCAGTGAGGCACCTATCTCAGCGATCTGTCTATTTCGTTCATCCATAGTTG
CCTGACTCCCCGTCGTGTAGATAACTACGATACGGGAGGGCTTACCATCTGGCCCCAGTGCTGCAATGATACCGCGAGAACCACGCTCACCG
GCTCCAGATTTATCAGCAATAAACCAGCCAGCCGGAAGGGCCGAGCGCAGAAGTGGTCCTGCAACTTTATCCGCCTCCATCCAGTCTATTAAT
TGTTGCCGGGAAGCTAGAGTAAGTAGTTCGCCAGTTAATAGTTTGCGCAACGTTGTTGCCATTGCTACAGGCATCGTGGTGTCACGCTCGTCG
TTTGGTATGGCTTCATTCAGCTCCGGTTCCCAACGATCAAGGCGAGTTACATGATCCCCCATGTTGTGCAAAAAAGCGGTTAGCTCCTTCGGT
CCTCCGATCGTTGTCAGAAGTAAGTTGGCCGCAGTGTTATCACTCATGGTTATGGCAGCACTGCATAATTCTCTTACTGTCATGCCATCCGTAA
GATGCTTTTCTGTGACTGGTGAGTACTCAACCAAGTCATTCTGAGAATAGTGTATGCGGCGACCGAGTTGCTCTTGCCCGGCGTCAATACGGG
ATAATACCGCGCCACATAGCAGAACTTTAAAAGTGCTCATCATTGGAAAACGTTCTTCGGGGCGAAAACTCTCAAGGATCTTACCGCTGTTGA
GATCCAGTTCGATGTAACCCACTCGTGCACCCAACTGATCTTCAGCATCTTTTACTTTCACCAGCGTTTCTGGGTGAGCAAAAACAGGAAGGC
AAAATGCCGCAAAAAAGGGAATAAGGGCGACACGGAAATGTTGAATACTCATACTCTTCCTTTTTCAATATTATTGAAGCATTTATCAGGGTT
ATTGTCTCATGAGCGGATACATATTTGAATGTATTTAGAAAAATAAACAAATAGGGGTTCCGCGCACATTTCCCCGAAAAGTGCCAC
```

Note: The underlined sequence indicates the *DMD* intron 43 target region.

Supplementary Table S5

Genome copies and functional vector particle titers in purified AdV preparations

AdV stock	Genome copies per ml (GC/ml)	Infectious units per ml (IU/ml)	GC/IU
AdV.gRNA^{S1}	9,44E+11	1,33E+11	7,10
AdV.Cas9	9,96E+11	1,39E+11	7,17
AdV.Cas9^{EX51}	1,04E+12	1,39E+11	7,48
AdV.Cas9^{EX53}	1,51E+12	2,77E+11	5,45
AdV.Cas9$^{IN52.IN53}$	1,03E+12	7,50E+10	13,73
AdV.Cas9$^{IN43.IN54}$	1,14E+12	1,17E+11	9,74

Adenoviral vectors encoding CRISPR/Cas9 multiplexes rescue dystrophin synthesis in unselected populations of DMD muscle cells

Ignazio Maggio, Jin Liu, Josephine M. Janssen, Xiaoyu Chen and Manuel A.F.V. Gonçalves

Supplementary Table S6

Primer pairs and composition for qPCR mixtures used for the quantification of genomic deletions.

Target	Primer code	Primers (5' → 3')	iQ SYBR Green supermix	Amplicon size (bp)
Intronic junction Δ53	#1190	TGCCTGGTATGTCTAGCCTG (150nM)	1 ×	128
	#1193	CCGAATGGTTGACTTCATGCA (150nM)		
Intronic junction Δ44-54	#1186	TGGTGTCATCCTGGAACTGC (150nM)	1 ×	99
	#1189	TCCACATGCCTACCAACATCT (150nM)		
Internal control *DMD* intron 43	#1198	TCCCAGCACTTTGAGAGACC (150nM)	1 ×	75
	#1199	TCCATGTTGCTCAGTCTGGT (150nM)		

Supplementary Table S7

qPCR cycling parameters used for the quantification of genomic deletions.

Target	Denaturation	Amplification			Melt curve analysis
		Denaturation	Annealing/Extension + plate read	# cycles	
Intronic junction Δ53	95 °C<br>5 min	95 °C<br>10 sec	65 °C<br>25 sec	40	55 to 95 °C (0.5 °C increment/step)
Intronic junction Δ44-54	95 °C<br>5 min	95 °C<br>10 sec	65 °C<br>30 sec	40	55 to 95 °C (0.5 °C increment/step)
Internal control *DMD* intron 43	95 °C<br>5 min	95 °C<br>10 sec	65.5 °C<br>30 sec	35	55 to 95 °C (0.5 °C increment/step)
